# *N*-(2,6-Dimethyl­phen­yl)-2,4-dimethyl­benzene­sulfonamide

**DOI:** 10.1107/S1600536811031102

**Published:** 2011-08-06

**Authors:** P. G. Nirmala, Sabine Foro, B. Thimme Gowda

**Affiliations:** a Department of Chemistry, Mangalore University, Mangalagangotri 574 199, Mangalore, India; b Institute of Materials Science, Darmstadt University of Technology, Petersenstrasse 23, D-64287 Darmstadt, Germany

## Abstract

Mol­ecules of the title compound, C_16_H_19_NO_2_S, are bent at the *S* atom with a C—SO_2_—NH—C torsion angle of −60.0 (2)°. The dihedral angle between the phenyl­sulfonyl and aniline rings is 41.7 (1)°. In the crystal, mol­ecules are packed into centrosymmetric dimers through pairs of N—H⋯O(S) hydrogen bonds.

## Related literature

For the preparation of the title compound, see: Savitha & Gowda (2006 ▶). For hydrogen-bonding modes of sulfonamides, see: Adsmond & Grant (2001 ▶). For studies on the effects of substituents on the structures and other aspects of *N*-(ar­yl)-amides, see: Arjunan *et al.* (2004 ▶); Gowda *et al.* (2000 ▶), on *N*-(ar­yl)-methane­sulfonamides, see: Gowda *et al.* (2007 ▶), on *N*-(ar­yl)-aryl­sulfonamides, see: Gelbrich *et al.* (2007 ▶); Nirmala *et al.* (2010 ▶); Perlovich *et al.* (2006 ▶), and on *N*-chloro-aryl­sulfonamides, see: Gowda *et al.* (2003 ▶).
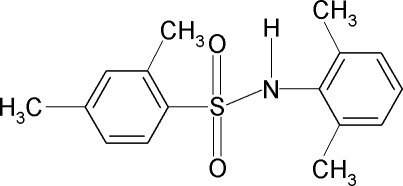


## Experimental

### 

#### Crystal data

C_16_H_19_NO_2_S
*M* *_r_* = 289.38
Monoclinic, 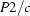

*a* = 11.119 (2) Å
*b* = 8.312 (1) Å
*c* = 16.043 (2) Å
β = 97.27 (1)°
*V* = 1470.8 (4) Å^3^
*Z* = 4
Mo *K*α radiation
μ = 0.22 mm^−1^
*T* = 293 K
0.48 × 0.36 × 0.20 mm

#### Data collection

Oxford Diffraction Xcalibur diffractometer with a Sapphire CCD detector
Absorption correction: multi-scan (*CrysAlis RED*; Oxford Diffraction, 2009 ▶) *T*_min_ = 0.901, *T*_max_ = 0.957
5256 measured reflections
2685 independent reflections
2107 reflections with *I* > 2σ(*I*)
*R*_int_ = 0.021

#### Refinement

*R*[*F* ^2^ > 2σ(*F* ^2^)] = 0.043
*wR*(*F* ^2^) = 0.125
*S* = 1.04
2685 reflections
188 parameters
1 restraint
H atoms treated by a mixture of independent and constrained refinement
Δρ_max_ = 0.28 e Å^−3^
Δρ_min_ = −0.28 e Å^−3^

### 

Data collection: *CrysAlis CCD* (Oxford Diffraction, 2009 ▶); cell refinement: *CrysAlis RED* (Oxford Diffraction, 2009 ▶); data reduction: *CrysAlis RED*; program(s) used to solve structure: *SHELXS97* (Sheldrick, 2008 ▶); program(s) used to refine structure: *SHELXL97* (Sheldrick, 2008 ▶); molecular graphics: *PLATON* (Spek, 2009 ▶); software used to prepare material for publication: *SHELXL97*.

## Supplementary Material

Crystal structure: contains datablock(s) I, global. DOI: 10.1107/S1600536811031102/bt5591sup1.cif

Structure factors: contains datablock(s) I. DOI: 10.1107/S1600536811031102/bt5591Isup2.hkl

Supplementary material file. DOI: 10.1107/S1600536811031102/bt5591Isup3.cml

Additional supplementary materials:  crystallographic information; 3D view; checkCIF report

## Figures and Tables

**Table 1:** Hydrogen-bond geometry (Å, °)

*D*—H⋯*A*	*D*—H	H⋯*A*	*D*⋯*A*	*D*—H⋯*A*
N1—H1*N*⋯O2^i^	0.83 (2)	2.20 (2)	3.024 (3)	168 (2)

Symmetry code: (i) 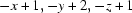.

**Fig. 1.**
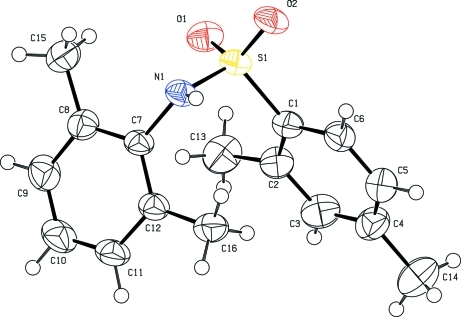
Molecular structure of the title compound, showing the atom labelling scheme and displacement ellipsoids are drawn at the 50% probability level.

**Fig. 2.**
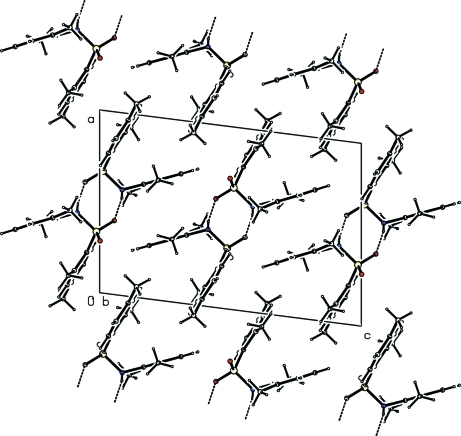
Molecular packing of (I) with hydrogen bonding shown as dashed lines.

## supplementary crystallographic information

### Comment

The amide and sulfonamide moieties are the constituents of many biologically important compounds. The hydrogen bonding preferences of sulfonamides have been studied (Adsmond & Grant, 2001). As part of our work on the effects of substituents on the structures and other aspects of *N*-(aryl)-amides (Arjunan *et al.*, 2004; Gowda *et al.*, 2000), *N*-(aryl)-methanesulfonamides (Gowda *et al.*, 2007), *N*-(aryl)-arylsulfonamides (Nirmala *et al.*, 2010) and *N*-chloro-arylsulfonamides (Gowda *et al.*, 2003), in the present work, the crystal structure of 2,4-dimethyl-*N*-(2,6-dimethylphenyl)benzenesulfonamide (I) has been determined (Fig. 1).

The N—C bond in the C—SO_2_—NH—C segment has *gauche* torsions with respect to the S═O bonds. The molecule is bent at the S atom with the C1—SO_2_—NH—C7 torsion angle of -60.0 (2)°, compared to the values of 70.1 (2) and -66.0 (2)° in the two independent molecules of 2,4-dimethyl-*N*-(2,3-dimethylphenyl)benzenesulfonamide (II), 66.5 (2)° in 2,4-dimethyl-*N*-(2,4-dimethylphenyl)-benzenesulfonamide (III), 53.9 (2)° in 2,4-dimethyl-*N*-(3,5-dimethylphenyl)- benzenesulfonamide (IV) and 46.1 (3)° (molecule 1) and 47.7 (3)° (molecule 2) in the two independent molecules of 2,4-dimethyl-*N*-(phenyl)- benzenesulfonamide (V)(Nirmala *et al.*, 2010).

The sulfonyl and the anilino benzene rings in (I) are tilted relative to each other by 41.7 (1)°, compared to the values of 41.5 (1) and 43.8 (1)° in the two molecules of (II), 41.0 (1)° in (III), 82.1 (1)° in (IV) and 67.5 (1)° (molecule 1) and 72.9 (1)° (molecule 2) in (V),

The remaining bond parameters in (I) are similar to those observed in (II), (III), (IV), (V) and other aryl sulfonamides (Perlovich *et al.*, 2006; Gelbrich *et al.*, 2007).

In the crystal, packing of molecules into chains through of N—H···O(S) hydrogen bonds (Table 1) is shown in Fig.2.

### Experimental

The solution of *m*-xylene (10 ml) in chloroform (40 ml) was treated dropwise with chlorosulfonic acid (25 ml) at 0 ° C. After the initial evolution of hydrogen chloride subsided, the reaction mixture was brought to room temperature and poured into crushed ice in a beaker. The chloroform layer was separated, washed with cold water and allowed to evaporate slowly. The residual 2,4-dimethylbenzenesulfonylchloride was treated with 2,6-dimethylaniline in the stoichiometric ratio and boiled for ten minutes. The reaction mixture was then cooled to room temperature and added to ice cold water (100 ml). The resultant solid 2,4-dimethyl-*N*- (2,6-dimethylphenyl)benzenesulfonamide was filtered under suction and washed thoroughly with cold water. It was then recrystallized to constant melting point from dilute ethanol. The purity of the compound was checked and characterized by recording its infrared and NMR spectra (Savitha & Gowda, 2006).

The prism like colourless single crystals used in X-ray diffraction studies were grown in ethanolic solution by a slow evaporation at room temperature.

### Refinement

The H atom of the NH group was located in a difference map and later restrained to the distance N—H = 0.86 (2) Å. The other H atoms were positioned with idealized geometry using a riding model with the aromatic C—H = 0.93Å and methyl C—H = 0.96 Å. The *U*_iso_(H) values were set at 1.2*U*_eq_(C-aromatic, N) and 1.5*U*_eq_(C-methyl)..

### Crystal data

**Table:**

C_16_H_19_NO_2_S	*F*(000) = 616
*M**_r_* = 289.38	*D*_x_ = 1.307 Mg m^−^^3^
Monoclinic, *P*2/*c*	Mo *K*α radiation, λ = 0.71073 Å
Hall symbol: -P 2yc	Cell parameters from 922 reflections
*a* = 11.119 (2) Å	θ = 2.5–27.7°
*b* = 8.312 (1) Å	µ = 0.22 mm^−^^1^
*c* = 16.043 (2) Å	*T* = 293 K
β = 97.27 (1)°	Prism, colourless
*V* = 1470.8 (4) Å^3^	0.48 × 0.36 × 0.20 mm
*Z* = 4	

### Data collection

**Table:**

Oxford Diffraction Xcalibur diffractometer with a Sapphire CCD detector	2685 independent reflections
Radiation source: fine-focus sealed tube	2107 reflections with *I* > 2σ(*I*)
graphite	*R*_int_ = 0.021
Rotation method data acquisition using ω and φ scans	θ_max_ = 25.4°, θ_min_ = 3.0°
Absorption correction: multi-scan (*CrysAlis RED*; Oxford Diffraction, 2009)	*h* = −12→13
*T*_min_ = 0.901, *T*_max_ = 0.957	*k* = −6→10
5256 measured reflections	*l* = −19→11

### Refinement

**Table:**

Refinement on *F*^2^	Primary atom site location: structure-invariant direct methods
Least-squares matrix: full	Secondary atom site location: difference Fourier map
*R*[*F*^2^ > 2σ(*F*^2^)] = 0.043	Hydrogen site location: inferred from neighbouring sites
*wR*(*F*^2^) = 0.125	H atoms treated by a mixture of independent and constrained refinement
*S* = 1.04	*w* = 1/[σ^2^(*F*_o_^2^) + (0.0674*P*)^2^ + 0.5567*P*] where *P* = (*F*_o_^2^ + 2*F*_c_^2^)/3
2685 reflections	(Δ/σ)_max_ = 0.005
188 parameters	Δρ_max_ = 0.28 e Å^−^^3^
1 restraint	Δρ_min_ = −0.28 e Å^−^^3^

### Special details

**Table:**

Geometry. All e.s.d.'s (except the e.s.d. in the dihedral angle between two l.s. planes) are estimated using the full covariance matrix. The cell e.s.d.'s are taken into account individually in the estimation of e.s.d.'s in distances, angles and torsion angles; correlations between e.s.d.'s in cell parameters are only used when they are defined by crystal symmetry. An approximate (isotropic) treatment of cell e.s.d.'s is used for estimating e.s.d.'s involving l.s. planes.
Refinement. Refinement of *F*^2^ against ALL reflections. The weighted *R*-factor *wR* and goodness of fit *S* are based on *F*^2^, conventional *R*-factors *R* are based on *F*, with *F* set to zero for negative *F*^2^. The threshold expression of *F*^2^ > σ(*F*^2^) is used only for calculating *R*-factors(gt) *etc*. and is not relevant to the choice of reflections for refinement. *R*-factors based on *F*^2^ are statistically about twice as large as those based on *F*, and *R*- factors based on ALL data will be even larger.

### Fractional atomic coordinates and isotropic or equivalent isotropic displacement parameters (Å^2^)

**Table:**

	*x*	*y*	*z*	*U*_iso_*/*U*_eq_	
C1	0.21154 (19)	0.9601 (3)	0.45025 (12)	0.0397 (5)	
C2	0.1037 (2)	0.9068 (3)	0.40278 (14)	0.0461 (6)	
C3	0.0200 (2)	1.0252 (3)	0.37507 (17)	0.0566 (7)	
H3	−0.0520	0.9933	0.3435	0.068*	
C4	0.0369 (2)	1.1871 (3)	0.39137 (16)	0.0541 (6)	
C5	0.1448 (2)	1.2349 (3)	0.43770 (15)	0.0529 (6)	
H5	0.1590	1.3434	0.4493	0.063*	
C6	0.2309 (2)	1.1225 (3)	0.46654 (14)	0.0444 (5)	
H6	0.3030	1.1559	0.4974	0.053*	
C7	0.37589 (17)	0.7507 (3)	0.33206 (12)	0.0355 (5)	
C8	0.37653 (19)	0.5848 (3)	0.31960 (14)	0.0432 (5)	
C9	0.3293 (2)	0.5267 (3)	0.24105 (15)	0.0540 (6)	
H9	0.3260	0.4163	0.2317	0.065*	
C10	0.2874 (2)	0.6298 (3)	0.17701 (15)	0.0563 (7)	
H10	0.2551	0.5888	0.1250	0.068*	
C11	0.2929 (2)	0.7931 (3)	0.18931 (14)	0.0484 (6)	
H11	0.2665	0.8615	0.1449	0.058*	
C12	0.33732 (19)	0.8582 (3)	0.26703 (13)	0.0400 (5)	
C13	0.0733 (2)	0.7344 (3)	0.38090 (19)	0.0631 (7)	
H13A	0.0683	0.6743	0.4315	0.076*	
H13B	0.1354	0.6892	0.3515	0.076*	
H13C	−0.0032	0.7295	0.3457	0.076*	
C14	−0.0588 (3)	1.3083 (4)	0.3596 (2)	0.0775 (9)	
H14A	−0.1099	1.3288	0.4024	0.093*	
H14B	−0.1068	1.2668	0.3104	0.093*	
H14C	−0.0205	1.4067	0.3458	0.093*	
C15	0.4295 (2)	0.4694 (3)	0.38653 (16)	0.0560 (6)	
H15A	0.3750	0.4581	0.4280	0.067*	
H15B	0.5060	0.5099	0.4126	0.067*	
H15C	0.4414	0.3665	0.3616	0.067*	
C16	0.3431 (2)	1.0378 (3)	0.27633 (15)	0.0517 (6)	
H16A	0.4105	1.0663	0.3171	0.062*	
H16B	0.2694	1.0761	0.2946	0.062*	
H16C	0.3532	1.0857	0.2232	0.062*	
N1	0.42138 (16)	0.8124 (2)	0.41422 (10)	0.0386 (4)	
H1N	0.466 (2)	0.894 (2)	0.4152 (15)	0.046*	
O1	0.28375 (16)	0.6738 (2)	0.50137 (10)	0.0517 (4)	
O2	0.40451 (14)	0.9080 (2)	0.55574 (9)	0.0496 (4)	
S1	0.33235 (5)	0.82853 (7)	0.48727 (3)	0.04002 (19)	

### Atomic displacement parameters (Å^2^)

**Table:**

	*U*^11^	*U*^22^	*U*^33^	*U*^12^	*U*^13^	*U*^23^
C1	0.0382 (11)	0.0462 (13)	0.0339 (11)	−0.0007 (10)	0.0014 (9)	0.0020 (9)
C2	0.0381 (12)	0.0505 (14)	0.0486 (13)	−0.0031 (11)	0.0011 (10)	0.0001 (11)
C3	0.0361 (12)	0.0683 (18)	0.0624 (15)	−0.0012 (12)	−0.0053 (11)	0.0030 (13)
C4	0.0458 (14)	0.0574 (16)	0.0592 (15)	0.0104 (12)	0.0067 (12)	0.0063 (12)
C5	0.0579 (15)	0.0475 (14)	0.0533 (14)	0.0033 (12)	0.0070 (12)	−0.0027 (11)
C6	0.0447 (12)	0.0478 (14)	0.0396 (12)	−0.0007 (11)	0.0006 (10)	−0.0038 (10)
C7	0.0288 (10)	0.0451 (12)	0.0306 (10)	0.0006 (9)	−0.0037 (8)	−0.0007 (9)
C8	0.0362 (11)	0.0452 (14)	0.0464 (12)	0.0010 (10)	−0.0016 (9)	−0.0017 (10)
C9	0.0522 (14)	0.0509 (15)	0.0562 (15)	−0.0032 (12)	−0.0036 (12)	−0.0148 (12)
C10	0.0496 (14)	0.0730 (18)	0.0425 (13)	−0.0029 (13)	−0.0091 (11)	−0.0152 (12)
C11	0.0434 (13)	0.0666 (17)	0.0327 (11)	0.0052 (12)	−0.0049 (10)	0.0016 (11)
C12	0.0347 (11)	0.0502 (14)	0.0341 (11)	0.0022 (10)	0.0004 (9)	0.0002 (9)
C13	0.0477 (14)	0.0585 (16)	0.0784 (18)	−0.0082 (13)	−0.0109 (13)	−0.0060 (14)
C14	0.0596 (17)	0.075 (2)	0.096 (2)	0.0187 (16)	0.0015 (16)	0.0111 (17)
C15	0.0622 (15)	0.0474 (14)	0.0562 (15)	0.0072 (13)	−0.0008 (12)	0.0045 (11)
C16	0.0613 (15)	0.0502 (15)	0.0425 (12)	0.0019 (12)	0.0027 (11)	0.0079 (11)
N1	0.0387 (10)	0.0431 (11)	0.0317 (9)	−0.0037 (8)	−0.0039 (7)	−0.0021 (8)
O1	0.0561 (10)	0.0492 (10)	0.0493 (9)	−0.0023 (8)	0.0044 (8)	0.0109 (7)
O2	0.0516 (9)	0.0622 (11)	0.0313 (8)	0.0006 (8)	−0.0089 (7)	−0.0041 (7)
S1	0.0414 (3)	0.0467 (3)	0.0300 (3)	−0.0006 (2)	−0.0033 (2)	0.0015 (2)

### Geometric parameters (Å, °)

**Table:**

C1—C6	1.387 (3)	C10—H10	0.9300
C1—C2	1.408 (3)	C11—C12	1.391 (3)
C1—S1	1.775 (2)	C11—H11	0.9300
C2—C3	1.388 (3)	C12—C16	1.500 (3)
C2—C13	1.504 (4)	C13—H13A	0.9600
C3—C4	1.379 (4)	C13—H13B	0.9600
C3—H3	0.9300	C13—H13C	0.9600
C4—C5	1.387 (4)	C14—H14A	0.9600
C4—C14	1.506 (4)	C14—H14B	0.9600
C5—C6	1.375 (3)	C14—H14C	0.9600
C5—H5	0.9300	C15—H15A	0.9600
C6—H6	0.9300	C15—H15B	0.9600
C7—C8	1.394 (3)	C15—H15C	0.9600
C7—C12	1.399 (3)	C16—H16A	0.9600
C7—N1	1.445 (2)	C16—H16B	0.9600
C8—C9	1.389 (3)	C16—H16C	0.9600
C8—C15	1.503 (3)	N1—S1	1.6325 (19)
C9—C10	1.373 (4)	N1—H1N	0.834 (16)
C9—H9	0.9300	O1—S1	1.4242 (17)
C10—C11	1.372 (4)	O2—S1	1.4364 (15)
			
C6—C1—C2	120.6 (2)	C7—C12—C16	123.77 (19)
C6—C1—S1	116.35 (17)	C2—C13—H13A	109.5
C2—C1—S1	122.97 (18)	C2—C13—H13B	109.5
C3—C2—C1	116.1 (2)	H13A—C13—H13B	109.5
C3—C2—C13	118.7 (2)	C2—C13—H13C	109.5
C1—C2—C13	125.2 (2)	H13A—C13—H13C	109.5
C4—C3—C2	124.2 (2)	H13B—C13—H13C	109.5
C4—C3—H3	117.9	C4—C14—H14A	109.5
C2—C3—H3	117.9	C4—C14—H14B	109.5
C3—C4—C5	117.9 (2)	H14A—C14—H14B	109.5
C3—C4—C14	121.0 (3)	C4—C14—H14C	109.5
C5—C4—C14	121.0 (3)	H14A—C14—H14C	109.5
C6—C5—C4	120.3 (2)	H14B—C14—H14C	109.5
C6—C5—H5	119.9	C8—C15—H15A	109.5
C4—C5—H5	119.9	C8—C15—H15B	109.5
C5—C6—C1	120.9 (2)	H15A—C15—H15B	109.5
C5—C6—H6	119.6	C8—C15—H15C	109.5
C1—C6—H6	119.6	H15A—C15—H15C	109.5
C8—C7—C12	122.12 (19)	H15B—C15—H15C	109.5
C8—C7—N1	118.32 (18)	C12—C16—H16A	109.5
C12—C7—N1	119.50 (19)	C12—C16—H16B	109.5
C9—C8—C7	117.8 (2)	H16A—C16—H16B	109.5
C9—C8—C15	119.7 (2)	C12—C16—H16C	109.5
C7—C8—C15	122.5 (2)	H16A—C16—H16C	109.5
C10—C9—C8	121.0 (2)	H16B—C16—H16C	109.5
C10—C9—H9	119.5	C7—N1—S1	120.67 (14)
C8—C9—H9	119.5	C7—N1—H1N	116.2 (16)
C11—C10—C9	120.3 (2)	S1—N1—H1N	109.3 (17)
C11—C10—H10	119.8	O1—S1—O2	118.69 (10)
C9—C10—H10	119.8	O1—S1—N1	108.46 (10)
C10—C11—C12	121.2 (2)	O2—S1—N1	104.84 (9)
C10—C11—H11	119.4	O1—S1—C1	108.85 (10)
C12—C11—H11	119.4	O2—S1—C1	107.37 (10)
C11—C12—C7	117.4 (2)	N1—S1—C1	108.20 (9)
C11—C12—C16	118.8 (2)		
			
C6—C1—C2—C3	−0.6 (3)	C8—C9—C10—C11	0.8 (4)
S1—C1—C2—C3	−177.08 (18)	C9—C10—C11—C12	−2.0 (4)
C6—C1—C2—C13	179.7 (2)	C10—C11—C12—C7	−0.1 (3)
S1—C1—C2—C13	3.2 (3)	C10—C11—C12—C16	179.0 (2)
C1—C2—C3—C4	−0.1 (4)	C8—C7—C12—C11	3.3 (3)
C13—C2—C3—C4	179.6 (3)	N1—C7—C12—C11	−179.25 (19)
C2—C3—C4—C5	0.7 (4)	C8—C7—C12—C16	−175.7 (2)
C2—C3—C4—C14	−179.5 (3)	N1—C7—C12—C16	1.7 (3)
C3—C4—C5—C6	−0.5 (4)	C8—C7—N1—S1	−86.7 (2)
C14—C4—C5—C6	179.7 (2)	C12—C7—N1—S1	95.8 (2)
C4—C5—C6—C1	−0.2 (4)	C7—N1—S1—O1	57.93 (19)
C2—C1—C6—C5	0.8 (3)	C7—N1—S1—O2	−174.33 (16)
S1—C1—C6—C5	177.48 (18)	C7—N1—S1—C1	−59.99 (19)
C12—C7—C8—C9	−4.4 (3)	C6—C1—S1—O1	153.48 (17)
N1—C7—C8—C9	178.15 (19)	C2—C1—S1—O1	−29.9 (2)
C12—C7—C8—C15	173.6 (2)	C6—C1—S1—O2	23.8 (2)
N1—C7—C8—C15	−3.8 (3)	C2—C1—S1—O2	−159.61 (18)
C7—C8—C9—C10	2.3 (4)	C6—C1—S1—N1	−88.85 (18)
C15—C8—C9—C10	−175.8 (2)	C2—C1—S1—N1	87.7 (2)

### Hydrogen-bond geometry (Å, °)

**Table:**

*D*—H···*A*	*D*—H	H···*A*	*D*···*A*	*D*—H···*A*
N1—H1N···O2^i^	0.83 (2)	2.20 (2)	3.024 (3)	168 (2)

Symmetry codes: (i) −*x*+1, −*y*+2, −*z*+1.
